# An SMS intervention to reduce caregiver’s sugar-sweetened beverages: impacts on theoretical constructs and parenting practices from a randomized controlled trial in rural appalachia

**DOI:** 10.1017/jns.2025.10057

**Published:** 2025-12-19

**Authors:** Brittany M. Kirkpatrick, Donna-Jean P. Brock, Annie L. Reid, Kathleen J. Porter, Theresa H. Markwalter, Wen You, Philip I. Chow, Lee Ritterband, Jamie Zoellner

**Affiliations:** 1 Department of Public Health Sciences, School of Medicine, UVA Cancer Center Research and Outreach Office, University of Virginia, Christiansburg, VA, USA; 2 Department of Public Health Sciences, School of Medicine, University of Virginia, Charlottesville, VA, USA; 3 Department of Psychiatry and Neurobehavioral Sciences, University of Virginia, Charlottesville, VA, USA

**Keywords:** Caregivers, Rural populations, Short message service, Sugar-sweetened beverages, Theoretical effectiveness, SMS, Short message service, SSB, Sugar-sweetened beverage, TPB, Theory of Planned Behaviour, IQR, Interquartile range

## Abstract

Kids SIP*smart*ER is a school-based behavioural intervention for rural Appalachia middle school students with an integrated two-way short message service (SMS) strategy for caregivers. When tested in a cluster randomized controlled trial, the intervention led to significant improvements in sugar-sweetened beverage (SSB) consumption among students and caregivers. This study explores changes in secondary caregiver outcomes, including changes in caregiver SSB-related theory of planned behaviour constructs (affective attitudes, instrumental attitudes, subjective norms, perceived behavioural control, and intentions), parenting practices, and the home environment. Participants included 220 caregivers (93% female, 88% White, 95% non-Hispanic, mean age 40.6) in Virginia and West Virginia at baseline and 7 months post-intervention. Relative to control caregivers (*n* = 102), intervention caregivers (*n* = 118) showed statistically significant improvements in instrumental attitudes (Coef.= 0.53, 95% CI [0.04, 1.01], *p* = 0.033), behavioural intentions (Coef.=0.46, 95% CI [0.05, 0.88], *p* = 0.027), parenting practices (Coef. = 0.22, 95% CI [0.11, 0.33], *p* < 0.001), and total home SSB availability (Coef. = –0.25, 95% CI [–0.39, –0.11], *p* < 0.001), with specific improvements for sweetened juice drinks (Coef. = –0.18, 95% CI [–0.35, –0.01], *p* = 0.043) and regular soda/soft drinks (Coef. = –0.31, 95% CI [–0.55, –0.07], *p* = 0.010). In contrast, there were no significant between group changes for affective attitudes, subjective norms, or perceived behavioural control. Our findings highlight future research areas and fill gaps in intervention literature. This study is among the few to develop and evaluate a scalable, theory-based caregiver SMS component in a rural, school-based intervention. Combined with evidence that Kids SIP*smart*ER improved SSB behaviours, our results emphasize the potential of theory-guided SMS interventions to impact SSB-related outcomes.

**Trial registration:** Clincialtrials.gov: NCT03740113.

## Introduction

Sugar-sweetened beverages (SSBs) are beverages with added sugar or other caloric sweeteners (i.e. high fructose corn syrup, sucrose, fruit juice concentrate, etc.).^(1)^ Examples include soda, fruit punch, lemonade, sports drinks, energy drinks, flavoured milk, and sweetened teas and coffees. Within the United States (US), SSBs account for the largest source of calories consumed, with 63% of adolescents and 49% of adults drinking at least 1 SSB per day.^(1)^ Surmounting evidence has shown a rise in non-communicable diseases (i.e. obesity, diabetes, cardiovascular diseases, dental caries, obesity-related cancers) associated with excessive SSB consumption.^(1,2)^ Notably, SSBs have been identified as a top contributor to the obesity epidemic, including linear dose-response associations between SSB consumption and obesity.^(3)^ Unfortunately, disparities in SSB consumption and related chronic conditions persist, including higher prevalence of SSB consumption in rural US regions such as Appalachia compared to more affluent suburban and urban US regions.^(4)^

Behavioural interventions that target multiple levels of the socioecological model have shown to be more effective at improving health behaviours like SSB intake; however, this type of approach is often underutilized.^(5)^ For example, evidence suggests that adolescents develop dietary behaviours based on their caregiver’s behaviour and home environment, as caregivers are both role models and gatekeepers of food and beverages within the home. The socioecological model is a framework used to understand the influence of multiple levels of the social environment including intrapersonal (i.e. knowledge, attitudes, self-efficacy), interpersonal (i.e. social networks, families), and environmental (i.e. home environment, community, policy) factors.^(6)^ Research shows that parenting practices, including restriction of an adolescent’s SSB intake, has been negatively associated with a child’s SSB consumption,^(7)^ while increased home availability has shown a positive association with SSB intake. Caregivers and adolescents consume 52% of calories from SSBs within the home, and adolescents have notably higher SSB consumption when their caregivers also consume SSBs.^(1,8)^ Thus, concerted efforts to prevent and reduce SSB intake among adolescents and their families include understanding caregiver’s SSB behaviours, how they structure the home environment, and their parenting practices around SSBs.^(9,10)^

While the socioecological model facilitates an understanding of what factors to target, the theory of planned behaviour (TPB) is useful for understanding how to change behaviours.^(11)^ The TPB is one of the most well-validated models for predicting health behaviours and has also been applied to the context of SSB reduction.^(12–15)^ It states that an individual’s behaviour arises from their behavioural intentions, which in turn are influenced by three related yet independent constructs: attitudes (i.e. emotions/feelings about a behaviour or evaluation of benefits/consequences of a behaviour), subjective norms (i.e. social expectations to adopt a behaviour), and perceived behavioural control (i.e. ease/difficulty to perform a behaviour).^(11)^ Existing research on caregiver SSB intake has identified constructs of the TPB to be the most prominent influences across the socioecological model, specifically, intake has shown to be inversely related to behavioural intentions, attitudes, and perceived behavioural control.^(13,16,17)^

Evidence shows that caregiver behaviours, parenting practices, and home availability are important interpersonal and environmental factors of socioecological model that can influence adolescent SSB intake.^(18–20)^ Several systematic reviews show that interventions targeting adolescent SSB intake often lack a caregiver component, and those that do vary widely in delivery.^(21–23)^ Interventions that include a caregiver component are rarely guided by socioecological models or behavioural theory, which may limit their effectiveness.^(22)^ Equally concerning, there are few multilevel SSB interventions with a caregiver component targeting rural areas.^(5)^ Furthermore, few known studies have used scalable and accessible means to deliver interventions, such as text message interventions to engage caregivers that has potential to impact the home environment.^(24–26)^ Theory-guided SMS interventions for non-caregivers have shown to be effective in improving health behaviours in the short-term,^(27,28)^ especially when utilizing personalized and tailored messages, but their long-term effects remain unclear. This points to important limitations in understanding how these theory-guided SMS interventions may effectively create and maintain healthier behaviours. With 94% of rural US owning a cell phone, there is tremendous opportunity to utilize SMS to initiate behaviour change in caregivers through interventions delivered to their mobile phones.^(29)^ However, few SMS behaviour interventions targeting SSB behaviours are conducted in rural areas,^(24)^ and these tend to be small-scale pilot or feasibility studies.^(25,26)^

This study is a cluster randomized control trial that was implemented in rural Appalachian middle schools.^(30)^ Schools were randomized to receive Kids SIP*smart*ER or to a control condition. Kids SIP*smart*ER is a 7-month school-based, behaviour and health literacy curriculum aimed at improving SSB behaviours among middle school students, which also utilizes an integrated two-way SMS strategy to engage caregivers to support SSB reductions at home. The purpose of the overall trial was to assess student and caregiver changes in SSB behaviours following intervention. Primary outcome findings provide evidence of significant improvements in SSB behaviours for both students and caregivers, relative to control participants. For caregivers specifically, regardless of SSB intake at baseline and relative to control caregivers, SSB significantly decreased among intervention caregivers.^(31)^ Process data from the SMS intervention also illustrate promising caregiver enrollment, retention, and engagement rates.^(32)^ However, the secondary caregiver outcomes, including postulated theoretical mechanisms of change in caregiver’s SSB behaviour, have not been explored and are the focus of this paper. Therefore, the primary aims of this study are to fill these gaps in knowledge by examining the impact of a theory-guided SMS intervention utilizing the socioecological model framework on improving caregivers’ SSB TPB constructs, parenting practices, and the home environment. We hypothesized that caregivers receiving the Kids SIP*smart*ER SMS intervention would show significant improvements in TPB constructs, parenting practices, and the home environment compared to control caregivers.

## Methods

### Design and setting

This trial includes 12 rural Appalachian schools (6 intervention and 6 control schools) from four counties in Virginia and one in West Virginia. Using simple randomization with each school year (SY) block, (i.e. Block 1:SY 2018-2019, Block 2:SY 2019-2020, Block 3:SY 2021-2022) four schools were randomized within each of the individual blocks (e.g. two schools randomized to the intervention condition and the other two to the control condition). Caregivers assigned to the intervention group received both the SMS intervention and survey assessments, whereas those in the control group only completed the survey assessments without receiving any SMS intervention content. Further details of the intervention protocol and primary outcomes of the larger type 1 hybrid design of this study have been previously published.^(30,31)^ This paper utilized caregivers’ secondary survey data from intervention and control schools. This study was conducted according to the guidelines laid down in the Declaration of Helsinki and all procedures involving research study participants were approved by the University of Virginia’s Institutional Review Board for the Social and Behavioural Sciences (IRB-SBS). Written or verbal informed consent was obtained from all caregivers, and assent was obtained from all student participants. Verbal consent was witnessed and formally recorded.

### Caregiver SMS intervention component

**Figure 1.**
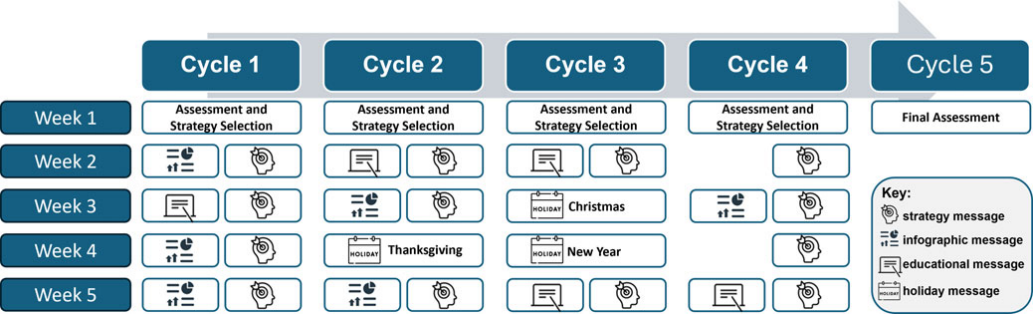
Sample caregiver SMS intervention process.

Development of the theory-guided caregiver SMS intervention was informed through formative research with caregiver stakeholders, who provided input on SMS messages and participated in a subsequent 5-week pilot trial.^(33)^ Findings from this formative phase led to the expansion and refinement of the current SMS intervention. First, caregivers received an initial newsletter highlighting the properties of SSBs, role modelling, and the home environment. As illustrated in Figure 1, caregivers received a baseline assessment message followed by four cycles of SMS messages. Caregivers who did not complete the baseline assessment were removed from the programme if they did not complete the second assessment at the beginning of cycle 2. Each cycle was four or five weeks long. Cycles started with a two-way assessment SMS message where caregivers would report their personal SSB intake, their child’s SSB intake, and then selected a strategy topic they wished to receive messages about over the cycle. If caregivers did not select a personalized strategy selection, they would receive a qualitative response strategy message for that cycle. Next, during the fall, caregivers received two weekly SMS messages: (1) a targeted educational message that paralleled the student in-class curriculum and (2) a strategy message (e.g. tips on breaking habits, overcoming taste, parenting, home/shopping, family and friends). Messages were sent biweekly in the spring. This SMS intervention was personalized by using the names of caregivers and their students within all assessment messages and many educational and strategy messages, as this has been found to increase intervention efficacy.^(27)^ After the baseline assessment, subsequent assessment messages provided additional personalization with a feedback statement on the caregiver and student’s progress since the last assessment. Additionally, strategy messages were mapped to theoretical constructs, such as tips on breaking habits and overcoming taste mapped primarily to TPB, parenting to parenting practices, home/shopping to home environment, and family and friends to subjective norms. Table 1 shows the SMS messages mapped to the theoretical framework and includes example SMS messages.

**Table 1.** Mapping the Kids SIPsmartER Short Message Service (SMS) Intervention Messages to TPB Constructs, Parenting Practices, and Home Environment

Theoretical Construct	Definition	Example Message
**Theory of Planned Behaviour**		
Affective Attitudes	The emotion and affect-based perceptions about SSBs (e.g. enjoyable or unenjoyable).	“KidsSIPsmartER: Sugary drinks include: soda/pop, sweet tea, sports drinks, juices, and energy drinks. Think about which ones you drink.” **[Week 2,** ***Educational*** **]** “KidsSIPsmartER: If at first you try a non-sugary drink and don’t like it, keep trying different ones. You’ll eventually find one that you like.” **[Week 12,** ***Strategy, <<Overcoming Taste>>*** **]**
Instrumental Attitudes	The perceptions of the costs and benefits of SSBs (e.g. healthy or unhealthy).	“KidsSIPsmartER: Sugary drink companies are good at using ads to convince us to buy their stuff. Don’t let these companies fool you!” **[Week 7,** ***Educational*** **]** “KidsSIPsmartER: During meal & snack times, offer your kids a variety of healthy drinks to choose from. Kids love having choices even if they are all healthy!” **[Week 3,** ***Strategy, <<Home or Shopping Tips>>*** **]**
Subjective Norms	One’s perception about the social expectations or how others would approve and disapprove of SSBs.	“KidsSIPsmartER: *Child’s Name* created a public service announcement about drinking fewer sugary drinks. Ask how s/he likes being a leader for a healthier community.” **[Week 12,** ***Educational*** **]** “KidsSIPsmartER: Share your goals related to reducing sugar drinks with your family and friends. Ask them to support you and hold you accountable.” [**Week 3,** ***Strategy, <<Family and Friends >>***]
Perceived Behavioural Control	Beliefs related to the perceived ease or difficulty of reducing SSB intake.	“KidsSIPsmartER: Set goals to stay on track! Having a goal and a plan to reach it can make it easier to drink fewer sugary drinks.” **[Week 15,** ***Educational*** **]** “KidsSIPsmartER: Wondering where to start breaking your habit? Try keeping a log of when and where you drink sugary drinks.” **[Week 1,** ***Strategy, <<Breaking Habit >>*** **]**
Behavioural Intention	A person’s relative strength of intention to reduce SSB intake. Intention is proposed to be predicted by the other TPB constructs: attitudes, subjective norms, and perceived behavioural control.	“KidsSIPsmartER: Congrats on making it this far! Keep up with your plans and if you slip up don’t give up! Ask *Child’s Name* about “No Guilt, No Giving Up!” **[Week 21,** ***Educational*** **]** “KidsSIPsmartER: If you are craving a sugary drink, stop and ask yourself if you really need it. Try waiting 20 minutes before deciding to have one or not.” **[Week 4,** ***Strategy, <<Overcoming Taste>>*** **]**
**Parenting Practices**		
Parenting Practices	Includes the totality of attitudes, values, beliefs, and behaviours that parents bring to settings in which they interact with their child about SSBs.	“KidsSIPsmartER: *Child’s Name* knows why and how to drink fewer sugary drinks. Encourage them to become a leader to help others drink less.” **[Week 10,** ***Educational*** **]** “KidsSIPsmartER: Make rules around sugary drinks. For example, try to limit *Child’s Name* sugary drinks to only special events, like parties.” **[Week 7,** ***Strategy, <<Parenting Strategies >>*** **]**
**Home Environment**		
Home Availability	The availability of SSB within the caregiver’s home.	“KidsSIPsmartER: Drink less, live more, throw sugar out the door! Limit sugary drinks to 8oz for adults and 0 for kids to improve health for the whole fam.” **[Week 3,** ***Educational*** **]** “KidsSIPsmartER: Keep kids hydrated by stocking your fridge with a variety of flavoured waters & fruits. This is much better than sugary drinks like Gatorade.” **[Week 13,** ***Strategy, <<Home or Shopping Tips>>*** **]**

### Eligibility and recruitment

One caregiver per household, regardless of SSB intake, was eligible to participate in Kids SIP*smart*ER by meeting the following eligibility criteria: legal guardian of a 7^th^-grade student at one of the twelve participating schools; ownership of a phone with text message capability; willingness to complete evaluation activities either by mail or through SMS.

Recruitment of caregivers was customized for each school and included recruitment packets (i.e. Kids SIP*smart*ER brochure, principal information letter, and consent form) delivered in a variety of recruitment methods such as included in back-to-school packets, delivered to students by their Health/PE teacher, completed as part of back-to-school or meet-the-teacher nights, etc. Consents were completed in three different formats: pen/paper, online through Qualtrics platform (QR code made available on principal letter), and/or through verbal consents obtained over the phone.

### Measures

Caregiver TPB constructs were assessed using the validated SSB-related TPB instrument.^(12,13)^ Survey items measured all five TPB constructs: affective attitude (1-item), instrumental attitude (1-item), subjective norms (1-item), perceived behavioural control (1-item), and behavioural intention (2-items). All items were assessed on a 7-point Likert scale [1 = Strongly disagree to 7 = Strongly agree]

A 10-item measure was developed to assess caregiver parenting practices from two different established instruments(i.e. the family and home-related factors scale and the Family Life, Activity, Sun, Health, and Eating Survey)^(34,35)^ and rescaled on consistent 5-point Likert scales [1=Strongly disagree 5= Strongly agree]. Examples of items include “I monitor how many SSBs my child has”; “There are rules in my home about how many SSBs my child can have”; and “It’s okay for me to make rules about how much sugar drinks my child can have”. A total score was calculated as the mean of the 10-items.^(34,35)^ The Cronbach’s α from our study sample was 0.68.

The SSB home environment was assessed by the reported home availability of five drink types (i.e. regular soft drinks, sweetened juice drinks, sweetened tea, coffee with sugar, energy/sports drinks) over the past month. Items were assessed on a 5-point Likert scale [1 = Never to 5 = Always], and total score was calculated as the mean of the items.^(35,36)^

Demographic characteristics were collected, including: (1) sex: male or female; (2) age: continuous; (3) race: White, Black, other; (4) ethnicity: Hispanic or non-Hispanic; (5) household income: four categories ranging from less than $25,000 to greater than $75,000 annually; (6) education: three categories of high school diploma/GED or less, some college/associate degree, or four-yr degree or more; (7) marital status: married/live-in partner or not marries/no live-in partner; (8) SNAP/WIC enrollment: yes or no (9) weight status: underweight, healthy weight, overweight, obese, severely obese.

### Statistical analyses

For this analysis of secondary caregiver variables, the completers analytic sample (as replicated from the primary SSB outcome analysis) were used.^(31)^ First, only caregivers with a completed consent form and both baseline and 7-month surveys were included in this analysis. Second, data were examined for the presence of outliers. Outliers were identified based on interquartile range (IQR) of the 0–7-month SSB change scores, and a conservative SSB change scores ≥ 2 × IQR were marked as outliers and excluded from analysis.^(37,38)^

Descriptive statistics were utilized to summarize caregiver demographics using SPSS version 27. To estimate the between-group treatment effects over time for caregiver TPB constructs, parenting practices, and the home environment, we used generalized linear models with a log link function and gamma distribution. These models were specified to account for time and group dummies, and their two-way interaction, which allowed us to capture the treatment effects over the 7-month period. We controlled for a set of a priori specified influencing factors, including gender, race, and year fixed effects, to ensure the validity of the estimated effects. Additionally, standard errors were adjusted to be robust to school-year cohort clustering, accounting for intra-school correlations in the data.^(37,38)^ Model analyses were conducted using Stata version 17.

## Results

### Participants

Of approximately 620 attending students from the 6 intervention schools, 190 (31%) caregivers enrolled in the intervention, and 126 (66%) completed the 7-month follow-up. Eight intervention caregivers were identified as outliers, resulting in 118 (62%) caregivers for analysis. Of approximately 802 attending students from the 6 control schools, 158 (20%) caregivers enrolled in the trial, and 110 (70%) completed the 7-month follow-up. Eight control caregivers were identified as outliers, resulting in an analytic sample of 102 (65%) caregivers. Attrition after randomization was primarily due to loss of follow-up and withdrawal from the study for both intervention and control caregivers.

As shown in Table 2, caregivers had a mean age of 40.6 (SD = 6.7) years old. Caregivers were primarily female (93%), White (88%), non-Hispanic (95%), and married or living with a romantic partner (79%). Related to socioeconomic variables, 34% of households had less than $50,000 annual income, 25% had educational attainment of a high school diploma or less, and 17% were receiving SNAP or WIC benefits. Additionally, caregivers’ BMI categorization included 20% healthy weight, 26% overweight, 19% obese, and 25% severely obese.

**Table 2.** Baseline demographics of caregivers included in the analysis, by randomized condition

Variable	Kids SIPsmartER *n* = 118	Control *n* = 102
	*n* (%)	*n* (%)
**Sex**		
Male	6 (5%)	6 (6%)
Female	111 (94%)	93 (91%)
Other/Unknown	1 (1%)	3 (3%)
**Age**		
≤ 32 years old	12 (10%)	7 (7%)
33−44 years old	71 (60%)	72 (71%)
≥ 45 years old	33 (28%)	19 (19%)
Unknown/Not reported	2 (2%)	4 (3%)
Mean	40.8 (7.0)	40.4 (6.2)
**Race**		
White	105 (89%)	88 (86%)
Black	3 (3%)	4 (4%)
Other	3 (3%)	5 (5%)
Unknown/Not reported	7 (5%)	5 (5%)
**Ethnicity**		
Hispanic	1 (1%)	0 (0%)
Not Hispanic	112 (99%)	96 (97%)
Not Sure	0 (0%)	3 (3%)
**Household Income**		
< $25,000	20 (17%)	18 (18%)
$25,000−$49,999	16 (14%)	20 (20%)
$50,000−$74,999	25 (22%)	18 (18%)
≥ $75,000	37 (31%)	31 (30%)
Unknown/Not reported	20 (16%)	15 (14%)
**Educational Attainment**		
High school, GED, or less	28 (24%)	28 (27%)
Some college/associate degree	49 (42%)	36 (35%)
4-year college degree or higher	36 (31%)	35 (34%)
Unknown/Not reported	5 (3%)	3 (4)
**Marital Status**		
Married/live-in partner	99 (84%)	75 (74%)
Not married/no live-in partner	18 (15%)	24 (24%)
Unknown/Not reported	1 (1%)	3 (2%)
**Receiving SNAP/WIC Benefits**		
Yes	16 (14%)	22 (22%)
No	96 (81%)	76 (75%)
Unknown/Not reported	6 (5%)	4 (3%)
**Weight Status**		
Underweight	1 (1%)	3 (3%)
Normal Weight	16 (14%)	28 (27%)
Overweight	31 (26%)	27 (26%)
Obese	26 (22%)	16 (16%)
Severely Obese	34 (29%)	21 (21%)
Unknown/Not reported	10 (8%)	7 (7%)

### TPB

Table 3 depicts changes in TPB constructs for caregivers over the 7-month period. Relative to the control condition, caregivers receiving the Kids SIP*smart*ER intervention had a statistically significant improvement for instrumental attitudes [0.53 units (95% CI = 0.04, 1.01); *p* = 0.033, effect size (ES) = 0.29] and for behavioural intentions [0.46 units (95% CI = 0.05, 0.88); *p* = 0.027, ES = 0.31]. In contrast, affective attitudes, subjective norms, and perceived behavioural control did not show statistically significant changes between conditions.

**Table 3.** Caregiver sugar sweetened beverages-related theory of planned behaviour constructs, parenting practices, and home environment outcomes by randomized condition

Variable	Sample size *n*	Kids SIP*smart*ER						Control						Relative effects between conditions^b^		
		Baseline^a^		7-month^a^		Adjusted change baseline to 7-month^b^		Baseline^a^		7- month^a^		Adjusted change baseline to 7-month^b^				
		Mean	SD	Mean	SD	Coeff (95% CI)	p-value	Mean	SD	Mean	SD	Coeff (95% CI)	p-value	Coeff (95% CI)	p-value	Effect size
**Caregiver SSB TPB Constructs** ^c^																
Affective Attitude	218	4.53	1.76	4.77	1.79	0.25 (0.03, 0.47)	0.026	4.49	1.77	4.57	1.71	0.07 (–0.22, 0.36)	0.627	0.18 (–0.18, 0.54)	0.323	0.13
Instrumental Attitude	216	5.50	1.55	5.95	1.51	0.44 (0.11, 0.78)	0.011	5.25	1.77	5.18	1.62	–0.08 (–0.43, 0.26)	0.638	0.53 (0.04, 1.01)	0.033	0.29
Subjective Norms	217	4.71	1.61	4.35	1.85	–0.35 (–0.59, –0.11)	0.005	4.08	1.77	4.06	1.75	–0.01 (–0.48, 0.45)	0.951	–0.34 (–0.87, 0.20)	0.215	0.18
Perceived Behavioural Control	217	6.08	1.54	5.84	1.85	–0.23 (–0.54, 0.08)	0.146	5.65	1.97	5.66	1.98	0.004 (–0.25, 0.26)	0.976	–0.24 (–0.63, 0.16)	0.245	0.15
Behavioural Intentions^d^	216	4.70	1.93	5.13	1.78	0.43 (0.15, 0.71)	0.003	4.45	2.27	4.42	2.10	–0.03 (–0.33, 0.26)	0.827	0.46 (0.05, 0.88)	0.027	0.31
**Caregiver SSB Parenting Practices** ^e^																
Parental Practices Total Score	216	3.61	0.60	3.79	0.57	0.18 (0.12, 0.24)	<0.000	3.70	0.63	3.66	0.57	–0.04 (–0.13, 0.05)	0.362	0.22 (0.11, 0.33)	0.000	0.56
**Caregiver SSB Home Environment** ^f^																
SSB Home Availability Total Score^g^	216	2.79	0.71	2.52	0.78	–0.27 (–0.35, –0.19)	<0.000	2.70	0.78	2.68	0.70	–0.02 (–0.13, 0.09)	0.734	–0.25 (–0.39, –0.11)	0.000	0.49
Sweetened juice drinks	216	2.64	1.13	2.43	1.12	–0.20 (–0.33, –0.06)	0.005	2.60	1.12	2.58	1.16	–0.02 (–0.13, 0.09)	0.773	–0.18 (–0.35, –0.01)	0.043	0.27
Regular soda or soft drinks	212	3.17	1.36	2.87	1.32	–0.30 (–0.39, –0.20)	<0.000	2.90	1.25	2.92	1.21	0.02 (–0.20, 0.24)	0.877	–0.31 (–0.55, –0.07)	0.010	0.37
Energy and sports drinks	214	2.44	1.35	2.08	1.14	–0.36 (–0.51, –0.22)	<0.000	2.42	1.19	2.26	1.21	–0.16 (–0.41, 0.09)	0.210	–0.21 (–0.50, 0.09)	0.165	0.20
Sweet tea (with sugar)	213	2.47	1.26	2.21	1.26	–0.28 (–0.49, –0.06)	0.011	2.55	1.20	2.54	1.33	–0.01 (–0.20, 0.18)	0.944	–0.27 (–0.55, 0.01)	0.063	0.25
Tea or coffee, with cream and/or sugar	214	3.20	1.62	3.04	1.64	–0.18 (–0.37, 0.004)	0.056	2.98	1.59	3.07	1.63	0.08 (–0.30, 0.47)	0.671	–0.26 (–0.70, 0.17)	0.229	0.17

SD, standard deviation; SSB, sugar-sweetened beverages; TPB, theory of planned behaviour.

a Means and Standard Deviations are not adjusted for covariates.

b Models control baseline covariates including gender and race. The models’ standard errors are adjusted to be school-year cohort cluster robust.

which is reflected in the 95% confidence intervals and p-values.

c Unit is 7-point Likert Scale (1-7), higher scores indicate more positive agreement.

d A continuous score was developed from the Mean of the 7-point Likert Scale for this 2-item question.

e Unit is 5-point Likert Scale (1-5), higher scores indicate more positive agreement.

f Unit is 5-point Likert Scale (1-5), higher scores indicate more home availability of that beverage.

g Caregiver SSB Home Environment was assessed by the reported home availability of five drink types (i.e. regular soft drinks, sweetened juice drinks, sweetened tea, coffee with sugar, energy/sports drinks) over the past month. Total SSB home availability was derived as a mean of all five drink types.

### Parenting practice

Relative to the control condition, caregivers receiving the Kids SIP*smart*ER intervention had a statistically significant improvement for the parenting practices total score [+0.22 units (95% CI = 0.11, 0.33), *p* < 0.001, ES = 0.56)] (Table 3).

### Home environment

Relative to the control condition, caregivers receiving the Kids SIP*smart*ER intervention had a statistically significant reduction for total home SSB availability [–0.25 units (95% CI = –0.39, –0.11), *p* < 0.001, ES = 0.49)](Table 3). Similarly, analyses of individual SSBs demonstrated significant relative between group effects in favor of the Kids SIP*smart*ER intervention for reducing home availability of sweetened juice drinks (–0.018, *p* = 0.043, ES = 0.27) and regular soda/soft drinks (–0.31, *p* = 0.010, ES = 0.37). Also, availability of energy and sports drinks (–0.36, *p* < 0.001), sweet tea with sugar (–0.28, *p* = 0.011) improved with the intervention condition, yet no significant between group effects were noted.

## Discussion

Findings from our study indicate that a 7-month theory-guided SMS intervention improved caregivers’ parenting practices and the home availability of SSBs. However, changes in the TPB constructs were mixed, with some but not all constructs demonstrating improvement in the intervention condition compared to the control condition. Nevertheless, when considered alongside the primary outcome findings showing improvements in caregivers’ SSB behaviours and weight,^(31)^ these results highlight that the SMS intervention can influence theoretical constructs and behaviours. Since prior literature demonstrates that adolescent SSB interventions incorporating a caregiver component are more successful in reducing SSB intake for both adolescents and their caregiver post-intervention^(21,22,39)^, our findings reveal an important modality to intervene with caregivers. Specifically, our study fills key intervention literature gaps by being one of the only studies to develop and evaluate a scalable and theory-guided caregiver SMS component for a rural, school-based SSB intervention.

In alignment with our study hypotheses, both parenting practices and home environment showed significant improvements in the Kids SIP*smart*ER intervention, relative to the control condition. Both parenting practices and the home environment showed medium effect sizes, highlighting the meaningfulness of these changes and the impact of the SMS intervention. These findings generally align with prior literature demonstrating intervention impacts on the home environment, parenting practices, and rulemaking around SSBs.^(21,22,33,34,39)^

In terms of the TPB constructs, the Kids SIPsmartER intervention group demonstrated significant improvements in instrumental attitudes and behavioural intentions compared to the control group. However, no significant differences were found in affective attitudes, subjective norms, or perceived behavioural control. It’s important to note that our exploratory study of secondary outcomes was not specifically powered to assess these theoretical effects, so the null findings should be interpreted with caution. Nonetheless, comparing these results with existing literature is valuable to identify both similarities and differences.^(40–43)^ Behavioural intentions are typically the most immediate predictor of actual behaviour and are often considered one of the strongest indicators of behaviour change, which aligns with our findings. However, perceived behavioural control is also regarded as a strong predictor of behaviour change, yet our study did not show improvements in this area, despite improvements in SSB behaviour. This is a notable discrepancy to the literature, including SSB-specific literature.^(13,16,17)^ Additionally, the mixed results in attitudes and subjective norms warrant further attention. Although the exact reasons for these mixed findings are unclear, other reviews of TPB-based behaviour change interventions, including those focused on nutrition, have also reported similar inconsistent effects on these constructs.^(40–43)^ As suggested by prior reviews, these variations may be partly attributed to the specific behaviour targeted in the intervention, as well as the unique socio-cultural and environmental context of rural Appalachian regions, where resources and opportunities to plan and control behaviours may be limited.^(44,45)^ Future adaptations of the SMS intervention could focus on modifying messages to better addresses and prioritize messages aimed at enhancing perceived behavioural control and subjective norms. Deviations from the TPB literature highlights the importance of considering socio-cultural and environmental factors when designing interventions. Despite our mixed findings, applying the TPB through all phases of the research – including alignment of intervention content, measurement and evaluation – was valuable and likely promoted the overall success of the intervention.

Theory-guided interventions are notably underrepresented in SMS-driven intervention literature, despite evidence that such approaches enhance effectiveness, particularly by facilitating behaviour change through improved caregiver constructs and adjustments to the home environment.^(21,22,39)^ Recent systematic reviews note that some of the greatest limitations in understanding how theoretical constructs may be applicable and effective within an SMS format stem from a lack of clear reporting on theoretical constructs utilized.^(27,28,46)^ Additionally, most SMS studies to date have focused on clinical care interventions, and those that have focused on behavioural changes have been statistically underpowered.^(28,46)^ Nutrition-related SMS interventions and systematic reviews have shown improvements in participant nutrition-related behaviours, but the understanding of how theory may be a factor in effectiveness is often not analysed or not clearly defined.^(47–49)^ A study conducted on improving parents’ intention to pack a healthy lunchbox and a systematic review on lifestyle changes for those with type 2 diabetes did address similar findings to our research on the importance of more theory-guided approaches in the development and measurement of SMS interventions.^(49,50)^ When evaluating SSB interventions, few acknowledge the utilization of theory in the development or implementation of the intervention and those that do often do not include measures for analysis of theoretical constructs.^(25,26,39)^ Our intervention design and findings are unique by adding to the literature that a theory-guided SMS intervention can be feasible and effective at improving caregiver’s SSB-related TPB constructs, parenting practices, and the home environment. Additionally, the small to medium effect sizes underline the meaningfulness of utilizing targeted theory-guided SMS interventions in improving SSB-related beliefs and behaviours.

Socio-demographic factors, including caregiver age, education, and income, were similar across the intervention and control groups, with a relatively homogeneous sample, primarily White and female. However, variations in these factors could influence caregivers’ engagement with and response to the intervention. Caregivers from different socio-economic backgrounds may have varying levels of access to resources and support, which could impact their participation and success. Future studies should compare these socio-demographic factors between groups to assess whether engagement or outcomes are affected by them. Additionally, exploring how socio-cultural norms in rural Appalachia shape nutrition-related caregiving behaviours could offer valuable insights into the broader applicability of these findings.

Our study findings may also have implications for public health policy, particularly in underserved rural areas. Integrating SMS interventions for caregivers of middle schoolers offers a scalable, cost-effective way to engage parents and improve health outcomes. Text messaging provides a low-barrier communication method, especially when traditional engagement is less accessible. This approach can enhance caregiver involvement in children’s health and behavioural changes, which is critical during the middle school years when habits are forming. While our study focused on reducing SSB, theory-driven SMS interventions may also be valuable in supporting other school wellness initiatives and programmes. In the context of public policy, incorporating SMS strategies into health interventions may support health equity by providing accessible support to parents with time or resource constraints, amplifying school-based health programmes. These opportunities are especially important given the barriers caregivers in rural areas face, including limited access to primary prevention interventions.^(40–42)^

Several study limitations should be considered when interpreting findings. First, all variables within our study were self-reported by caregivers and could be subject to self-report bias. Second, due to the targeted rural status and socio-cultural norms in this Appalachian region related to SSB behaviours, generalizability to other regions may be lacking. Third, as previously mentioned, this study was not specifically powered to examine these secondary effects, so null findings need to be cautiously interpreted. Fourth, since we are reporting secondary outcomes, and not primary outcomes, we did not apply multiplicity adjustment in the interpretation of the p-values. However, our finding focused on specific comparison of each secondary outcome between groups and the ‘per-comparison-wise error rate’ does not increase with multiple testing.^(51,52)^ Fifth, our retention data was impacted by COVID-19 related school closures. Because we cannot assume data is missing at random and since we are reporting secondary outcome variables, we did not apply intent-to-treat analytical procedures. Importantly, the demographic characteristics of caregivers retained for analysis in this study do not differ meaningfully from those of the originally enrolled sample.^(31)^ Finally, the calculated Cronbach’s alpha values for parenting practices in our sample were somewhat lower than the typical threshold of 0.8. Future research should focus on refining this measure to enhance reliability, particularly in rural contexts where behaviours may be shaped by distinct parenting, contextual, and environmental factors.

These limitations are balanced by the study strengths, including a strong theoretical framework, an RCT design, the use of validated questionnaires, and a focus on underserved rural populations. Additionally, while this study concentrated on secondary caregiver outcomes, the inclusion of a classroom-based behavioural intervention for students and an integrated SMS strategy for caregivers is another key strength. Further research is needed to explore the bidirectional relationship between caregivers’ and adolescents’ behaviours, such as how caregivers’ SSB consumption and decisions influence adolescents’ beliefs and behaviours, and vice versa. Understanding how targeted dyadic SSB interventions can enhance outcomes for both adolescents and caregivers is also crucial for improving effectiveness of nutrition-related, school-based interventions.

Our findings also highlight several other areas of future research. Additional studies with sufficient sample sizes are needed to understand if changes in caregiver’s SSB TPB constructs, parenting practices, and the home environment mediate changes in SSB behaviours. Furthermore, more long-term SMS interventions are needed to understand the ability of these interventions to not only improve SSB behaviours but maintain these changes. Finally, future research needs to adequately address the theoretical constructs utilized in the development and implementation of SMS interventions, as well as measure and analyse these theories in relation to the efficacy of the intervention.

## Conclusion

SMS interventions have become a more frequently utilized modality in delivering behavioural interventions. Our 7-month caregiver SMS intervention was effective at improving instrumental attitudes, behavioural intentions, parenting practices, and home availability of SSBs. When combined with primary outcome findings that demonstrate improvements in caregivers’ SSB behaviours, findings emphasize the promise of using a theory-guided brief SMS programme to reduce SSB intake among caregivers. Optimizing theory-guided SMS interventions in future research could maximize the potential effects of improving health disparities for rural caregivers. Additionally, SMS interventions are well-suited for rural Appalachian caregivers due to their accessibility, low cost, and scalability.
